# Dermatophyte abscesses caused by *Trichophyton rubrum* in a patient without pre-existing superficial dermatophytosis: a case report

**DOI:** 10.1186/s12879-016-1631-y

**Published:** 2016-06-17

**Authors:** Si-Hyun Kim, Ik Hyun Jo, Jun Kang, Sun Young Joo, Jung-Hyun Choi

**Affiliations:** Division of Infectious Diseases, Department of Internal Medicine, College of Medicine, The Catholic University of Korea, Incheon St. Mary’s Hospital, 56 Dongsu-ro, Bupyeong-gu, Incheon, 403-720 Republic of Korea; Department of Pathology, College of Medicine, The Catholic University of Korea, Incheon St. Mary’s hospital, Incheon, Republic of Korea; Department of Orthopedic Surgery, College of Medicine, The Catholic University of Korea, Incheon St. Mary’s Hospital, Incheon, Republic of Korea

**Keywords:** Trichophyton, Dermatophyte, Abscess, Soft tissue

## Abstract

**Background:**

*Trichophyton* usually causes a superficial skin infection, affecting the outermost layer of the epidermis, the stratum corneum. In immunocompromised patients, deeper invasion into the dermis and even severe systemic infection with distant organ involvement can occur. Most cases of deeper dermal dermatophytosis described in the literature so far involved pre-existing superficial dermatophytosis.

**Case presentation:**

We report a 68-year-old woman presented to our clinic with a 3-month history of palpable nodules on the right ankle without pre-existing superficial dermatophytosis. Magnetic resonance imaging showed multiple, well-demarcated, cystic lesions around the lateral malleolus, located in the subcutaneous or dermal layers. The sizes varied from 0.5 cm to 4 cm in diameter. The patient underwent complete excision of the lesions. Fungal culture yielded *Trichophyton rubrum* on Sabouraud dextrose agar. Histopathology showed organizing abscesses with degenerated fungal hyphae. After the 12-week oral itraconazole therapy, the lesions were completely resolved.

**Conclusion:**

Dermatophytes should be considered as a possible cause of deep soft tissue abscesses in immunocompromised patients, even though there is no superficial dermatophytosis lesion.

## Background

*Trichophyton* is a dermatophytic fungus, which is often responsible for nail, hair follicle, and superficial skin infections [1, 2]. In rare cases, it can cause a dermatophyte abscess in deeper skin layers or even disseminate to internal organs, including the lymph nodes, brain, liver, muscle, and bone. This can happen particularly in human immunodeficiency virus-infected or other immunocompromised patients [3–5]. The deep lesions are usually accompanied by superficial dermatophytosis.

Here, we describe a case of recurrent, multiple, dermatophyte abscesses caused by *Trichophyton rubrum* in an immunocompromised patient without pre-existing superficial dermatophytosis.

## Case presentation

A 68-year-old woman presented with a 3-month history of recurrent, multiple, subcutaneous nodules on the right ankle. Seven months previously, she had undergone complete excision of similar lesions at nearly the same sites at another hospital. Pathological examination had shown multiple abscesses with many fungal hyphae, but antifungal therapy was not prescribed. For the previous 7 years, she had been on medication for type 2 diabetes mellitus and hypertension, and for the previous 5 years, she had additionally been taking a daily dose of 10 mg prednisolone to treat psoriasis.

**Fig. 1:**
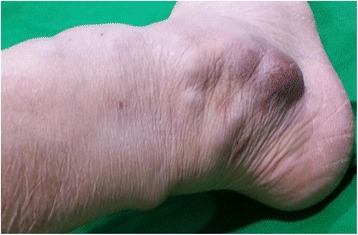
Clinical photograph of the right ankle before the operation

**Fig. 2:**
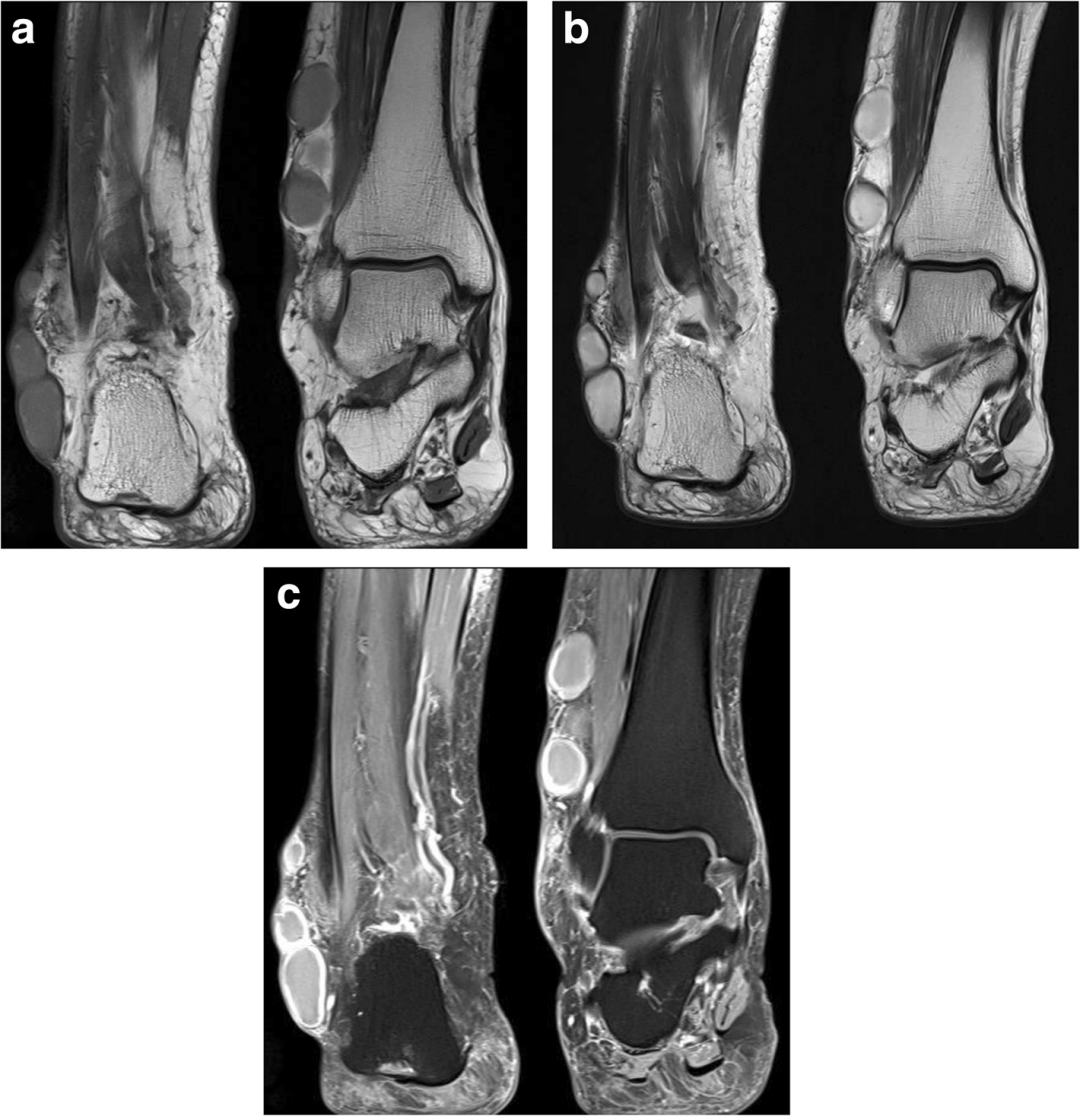
Magnetic resonance imaging of the right ankle at admission: (**a**) T1-weighted, (**b**) T2-weighted, and (**c**) fat-suppressed T1-weighted images

On the first visit to the orthopedic outpatient clinic, the patient’s vital signs were stable, with a blood pressure of 130/70 mmHg, pulse rate of 86 beats per minute, and body temperature of 36.2 °C. Physical examination showed multiple nodules located on the lateral peri-malleolar area and posterior aspect of the right ankle (Fig. 1). They were round, well-demarcated, and firmly palpable with fluctuation. The sizes varied from 0.5 cm to 4 cm in diameter. The patient did not report any itching, tenderness, or paresthesia on the lesion sites. There was no abnormal change in the skin and nails. Laboratory tests revealed the following: white blood cell count, 8,800/mm^3^ with 69.6 % segment neutrophils (normal, 4,000–9,900/mm^3^ with 39–72 % segment neutrophils); hemoglobin level, 13.8 g/dL (normal, 12–15 g/dL); platelet count, 231,000/mm^3^ (normal, 140,000–400,000/mm^3^); fasting glucose level, 147 mg/dL (normal, 75–115 mg/dL); and C-reactive protein level, 3.61 mg/L (normal, 0–5 mg/L). None of the blood chemistry data were significantly abnormal. Magnetic resonance imaging of the right ankle showed multiple, well-demarcated, cystic lesions around the lateral malleolus, located in the subcutaneous or dermal layers. The lesions had an intermediate signal intensity on T1-weighted images (Fig. 2a) and a high signal intensity on T2-weighted images (Fig. 2b), and showed rim enhancement after contrast medium injection on fat-suppressed T1-weighted images (Fig. 2c). The thick, purulent, exudative fluid obtained from the lesions by direct needle aspiration was examined by Gram staining, bacterial and fungal culture, acid-fast bacilli staining, mycobacterial culture, and real-time polymerase chain reaction assay to detect *Mycobacterium tuberculosis* and non-tuberculous mycobacteria. The patient was treated empirically with oral cefixime 100 mg twice daily for 14 days, but the lesions did not improve. After 18 days of incubation, the microbiology laboratory reported a slight growth of *Trichophyton rubrum* on Sabouraud dextrose agar. Results of other tests were all negative.

**Fig. 3:**
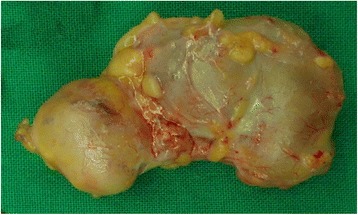
Excised surgical specimen of the abscess

**Fig. 4:**
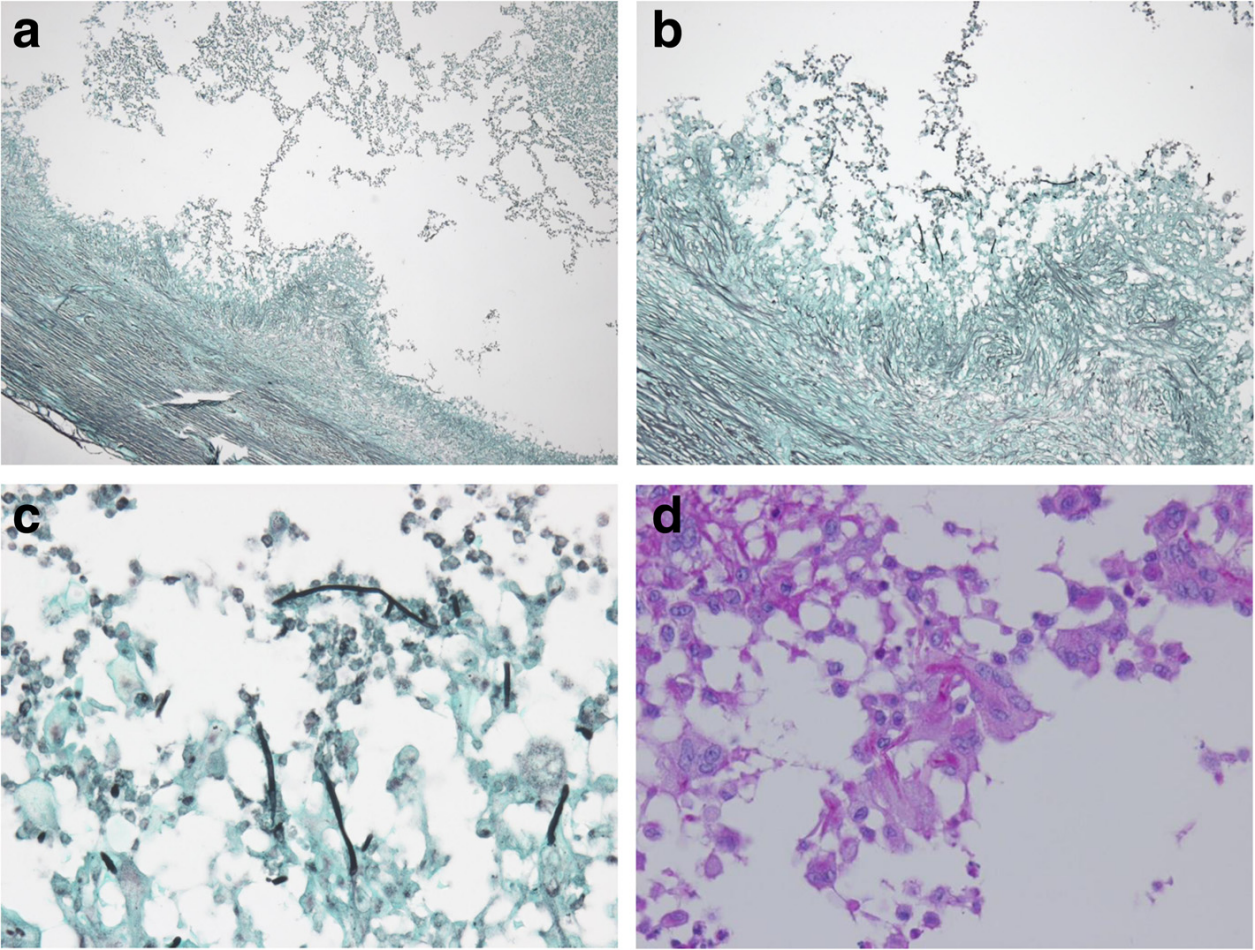
Histopathology of the surgical specimen show the hyaline, branching septate hyphae in multinucleated giant cells with surrounding neutrophils. (**a**–**c**) Grocott’s methenamine silver stain (×40, ×100, ×400) and (**d**) Periodic acid-Schiff stain (×400)

The patient was admitted to the hospital for complete excision and biopsy of the lesions. The operative findings were multiple, well-defined, multilobular nodules filled with pus, which were completely excised (Fig. 3). Bacterial and fungal culture of the pus and histopathology of the tissue samples were performed to discriminate true fungal infection from contamination. Histopathology showed organizing abscesses with degenerated fungal hyphae identified by Grocott's methenamine silver stain and periodic acid-Schiff stain (Fig. 4). Bacterial culture of the pus was negative and fungal culture yielded *Trichophyton rubrum*.

The patient was diagnosed with recurrent dermatophyte abscesses caused by *Trichophyton rubrum*, and oral itraconazole 200 mg once daily was started. Oral prednisolone slowly tapered and stopped 3 weeks after operation. She was discharged without any postoperative complications and prescribed continued oral itraconazole. After the 12-week oral itraconazole treatment course, the surgical wound remained clean and there was no evidence of the development of new lesions. A follow-up magnetic resonance imaging of the same site also showed no remnant abscess or any sign of recurrence. The patient did not show any recurrence at the 2.5 years follow-up.

## Discussion

Dermatophytes are fungi that commonly cause skin disease in humans and animals worldwide. Three types of dermatophytes, *Trichophyton*, *Microsporum*, and *Epidermophyton,* account for the majority of infections, which share similar pathognomonic characteristics; these are commonly referred to as ringworm [2]. *Trichophyton rubrum* is the most prevalent causative agent of human dermatophytosis, affecting nearly 90 % of patients with a dermatophyte infection in Korea [6, 7]. The infection can involve any area of the skin, but the most frequently affected sites are the feet (tinea pedis), groin (tinea cruris), scalp (tinea capitis), and nails (tinea unguium) [3].

These dermatophyte fungi typically attack the skin, hair, and nails because they require keratin for growth. In immunocompetent hosts, they cannot penetrate deeper than the stratum granulosum and their colonies are not usually observed in the layer over the epidermis. However, deeper dermis and subcutaneous dermatophyte infections can occur in patients with compromised immune systems, caused by solid organ transplantation, hematological malignancy, immunosuppressive therapy, or congenital immune deficiency [8, 9]. In these cases, the fungi can enter the bloodstream and disseminate to distant major organs, including the lymph nodes, liver, brain, and bone. This often causes systemic infections, which can be fatal [3, 10, 11].

A recent article provided a descriptive review of 46 cases of dermatophyte abscess [5]. Similar to our case, 38 patients (83 %) were immunocompromised, including those with solid organ transplantation, hematological malignancy, collagen disease, nephrotic syndrome, bullous pemphigoid, and diabetes mellitus. The majority of patients (83 %) presented with multiple nodules, and the lower extremities were most frequently affected (54 %). Most patients had pre-existing superficial dermatophytosis. More specifically, 12 of 14 cases (86 %) reported after 1999 involved superficial dermatophytosis (information was not available in the other 2 cases). In our case, however, there was neither a concomitant lesion nor a previous history of superficial dermatophytosis at any site of the body. Therefore, it was more difficult to make a definitive diagnosis.

The standard treatment for a dermatophyte abscess is not well established. In a recent article, various therapies were reported to have been used [5]. Most patients received systemic antifungal therapy, including amphotericin B, griseofulvin, fluconazole, itraconazole, micafungin, and terbinafine. Two patients were successfully treated with combined antifungal therapy and surgical excision. Colwell et al. reported a case of a dermatophyte abscess on the scalp caused by *Microsporum canis*, which was resolved after surgical excision without any antifungal therapy [12]. In contrast, the lesions in our case recurred several months after complete excision. Because the pathogenesis of deep dermatophytosis is poorly understood, we cannot fully explain the recurrence. However, there are a few possible explanations: (1) a difference in the causative fungi (*M. canis* vs. *Trichophyton* species); (2) a difference in the portal of entry of the fungi based on the presence of superficial dermatophytosis (data not available vs. no superficial dermatophytosis); and (3) a difference in the mode of spread inferred from the number of lesions (1 vs. multiple). In addition, the appropriate duration of antifungal therapy for a dermatophyte abscess is not yet established. Treatment should be according the severity of infection. Our patient was treated with itraconazole 200 mg daily for 12 weeks, the schedule commonly used in the treatment of tinea unguium.

## Conclusion

We report a rare case of dermatophyte abscesses caused by *Trichophyton rubrum* in an immunocompromised patient without pre-existing superficial dermatophytosis. A lack of experience with dermatophytosis could make clinicians underestimate the significance of positive dermatophyte fungal cultures obtained from deep soft tissue. Even without any superficial dermatophytosis lesion, fungi should be considered as a possible cause of deep soft tissue abscesses in immunocompromised patients, and a culture for fungi as well as bacteria should be performed in these patients.

## Abbreviations

Not applicable.

## References

[1] Havlickova B, Czaika VA, Friedrich M (2008). Epidemiological trends in skin mycoses worldwide. Mycoses.

[2] Seebacher C, Bouchara JP, Mignon B (2008). Updates on the epidemiology of dermatophyte infections. Mycopathologia.

[3] Dan P, Rawi R, Hanna S, Reuven B (2011). Invasive cutaneous *Trichophyton shoenleinii* infection in an immunosuppressed patient. Int J Dermatol.

[4] Seckin D, Arikan S, Haberal M (2004). Deep dermatophytosis caused by *Trichophyton rubrum* with concomitant disseminated nocardiosis in a renal transplant recipient. J Am Acad Dermatol.

[5] Inaoki M, Nishijima C, Miyake M, Asaka T, Hasegawa Y, Anzawa K, Mochizuki T (2015). Case of dermatophyte abscess caused by *Trichophyton rubrum*: a case report and review of the literature. Mycoses.

[6] Vena GA, Chieco P, Posa F, Garofalo A, Bosco A, Cassano N (2012). Epidemiology of dermatophytoses: retrospective analysis from 2005 to 2010 and comparison with previous data from 1975. New Microbiol.

[7] Lee WJ, Kim SL, Jang YH, Lee SJ, Kim Do W, Bang YJ, Jun JB (2015). Increasing prevalence of *Trichophyton rubrum* identified through an analysis of 115,846 cases over the last 37 years. J Korean Med Sci.

[8] Lillis JV, Dawson ES, Chang R, White CR (2010). Disseminated dermal *Trichophyton rubrum* infection - an expression of dermatophyte dimorphism?. J Cutan Pathol.

[9] Warycha MA, Leger M, Tzu J, Kamino H, Stein J (2011). Deep dermatophytosis caused by *Trichophyton rubrum*. Dermatol. Online J.

[10] Allen DE, Snyderman R, Meadows L, Pinnell SR (1977). Generalized *microsporum audoninii* infection and depressed cellular immunity associated with a missing plasma factor required for lymphocyte blastogenesis. Am J Med.

[11] Cheikhrouhou F, Makni F, Masmoudi A, Sellami A, Turki H, Ayadi A (2010). A fatal case of dermatomycoses with retropharyngeal abscess. Ann Dermatol Venereol.

[12] Colwell AS, Kwaan MR, Orgill DP (2004). Dermatophytic pseudomycetoma of the scalp. Plast Reconstr Surg.

